# Italian adaptation of the Düsseldorf Orthorexia Scale (I-DOS): psychometric properties and prevalence of orthorexia nervosa among an Italian sample

**DOI:** 10.1007/s40519-021-01278-2

**Published:** 2021-08-05

**Authors:** Silvia Cerolini, Mariacarolina Vacca, Andrea Zagaria, Lorenzo Maria Donini, Claudio Barbaranelli, Caterina Lombardo

**Affiliations:** 1 Department of Psychology, Sapienza University of Rome, via dei Marsi 78, 00185 Rome, Italy; 2 Department of Experimental Medicine, Medical Pathophysiology, Food Science and Endocrinology Section, Food Science and Human Nutrition Research Unit, Sapienza University of Rome, Piazzale Aldo Moro, Rome, Italy

**Keywords:** Orthorexia, Healthy eating, Scale validation, Confirmatory analysis, Psychometric properties

## Abstract

**Purpose:**

Orthorexia Nervosa (ON) is described as an extreme level of preoccupation around healthy eating, accompanied by restrictive eating behaviors. During the years, different assessment instruments have been developed. The aim of the study is to adapt into Italian the Düsseldorf Orthorexia Scale (I-DOS) and to test its psychometric properties.

**Method:**

A total sample of 422 volunteer university students (mean age = 20.70 ± 3.44, women 71.8%) completed a group of self-report questionnaires in large group sessions during their lecture time. The scales assessed ON (the I-DOS and the Orhto-15), disordered eating (Disordered Eating Questionnaire, DEQ), depressive symptoms (Beck Depression Inventory-II, BDI-II), obsessive and compulsive symptoms (Obsessive Compulsive Inventory-Revised, OCI-R), and self-reported height and weight.

**Results:**

The fit of the unidimensional structure and reliability of the I-DOS was tested trough Confirmatory Factor Analysis (CFA) as well as its criterion validity computing correlation coefficients among Ortho-15, DEQ, BDI-II, OCI-R, BMI. Analyses confirmed the unidimensional structure of the I-DOS with acceptable or great fit indices (CFI = 0.984; TLI = 0.978; SRMR = 0.043; RMSEA = 0.076) and the strong internal consistency (α = 0.888). The correlations path supported the criterion validity of the scale. The estimated total prevalence of both ON and ON risk was 8.1%.

**Conclusions:**

This 10-item scale appears to be a valid and reliable measure to assess orthorexic behaviors and attitudes.

**Level of evidence:**

Level V, descriptive cross-sectional study.

## Introduction

In the 90’s Bratman [1] described for the first time Orthorexia Nervosa (ON) as an extreme level of preoccupation around healthy eating, accompanied by restrictive eating behaviors. The term comes from the Greek “orthos” (correct, right) and “orexia” (appetite, hunger). The portmanteau term thus describes an excessive concern related to eating healthy foods to avoid adverse health outcomes [2]. Bratman considered it to be a disorder to the extent that the pursuit of healthy foods negatively impacted upon other areas of life such as work and relationships and was impairing and associated with significant changes in lifestyle [2, 3], as it is also associated to the reluctance to eat outside to avoid eating certain types of foods considered unhealthy, extreme preoccupation around eating only organic or “pure” foods, and excessive concern related to food quality [4].

Although the research on ON is currently flourishing, several limitations have been highlighted by a narrative review of the literature by Cena and colleagues [5], which accurately explores the main features and the problems sill related to this construct such as the terms used to describe and define ON and healthy eating, and the definition of clear and shared diagnostic criteria.

Despite the lack of consensus among the definition of ON by different authors, and the fact that it has not yet been included in the Diagnostic and Statistical Manual of Mental Disorders (DSM-5) [6], several instruments assessing this construct have been developed over the years [7].

Recently, Meule and colleagues [7] compared four of the most popular self-report scales for measuring ON: Bratman's Orthorexia Test (BOT) [2], the ORTO-15 [3], the Eating Habits Questionnaire (EHQ) [8], and the Düsseldorf Orthorexia Scale (DOS) [9], examining their factor structure, internal reliability, and the intercorrelations between them. Three of these scales (BOT, EHQ, and DOS) demonstrated to be valid and reliable instruments to assess orthorexia nervosa and the high intercorrelations across them (rs > 0.70) indicated that they essentially measure the same construct [10, 11]. Furthermore, in order to overcome the statistical limitations of the ORTO-15 [3] a revised version of this scale has been recently developed, the ORTO-R [12].

The aim of the present study is to adapt the Italian version of the Düsseldorf Orthorexia Scale (I-DOS) and to test its psychometric properties. The original German version of the scale was developed in 2015, but it was quickly adapted into English [13], Chinese [14], Spanish [15], Portuguese [16], Polish [17], and recently French [18]. The results from these studies converge in evidencing the good fit of the one factor structure of the scale and its good internal consistency (ranging between α = 0.84 to 0.88). In the present study, it was adapted into Italian and its psychometric properties were tested through Confirmatory Factor Analysis and correlations with other scales.

## Method

### Participants and procedure

A total sample of 422 young adults (mean age = 20.70 ± 3.44, women 71.8%) were recruited among the student community of Sapienza University of Rome, using a convenience sampling procedure. Both graduate or undergraduate students volunteered to participate in the study and provided a written informed consent. All participants completed a battery of self-report questionnaires in large group sessions during their lecture time between September 2019 and January 2020. The session lasted about 30 min. The study was approved by the Institutional Review Board of the Department of Psychology at Sapienza University of Rome.

### Measures

ON was measured using the I-DOS and the most widely used ORTO-15 [3], thus testing the convergent validity: (a) The Düsseldorfer Orthorexie Skala (DOS) [9]—the German version demonstrated good psychometric properties, with Cronbach α = 0.84 and test–retest reliability, r = 0.79. The scale (I-DOS) was translated and back-translated [19] and psychometric properties assessed. The translation phase involved three steps: an initial translation to Italian; a back-translation of the Italian version to the German version; a comparison of the original DOS scale with the back-translated version [20, 21]. Cronbach’s α of the Italian translation was 0.888, showing a strong internal consistency. The scale consists of 10 items assessing orthorexic behaviors and attitudes using a 4 a four-point Likert-scale from “this applies to me” (4 points) to “this does not apply to me” (1 point). The maximum score is 40 points and higher scores indicate more pronounced orthorexic behavior. A cutoff score of ≥ 30 indicates the presence of ON, while a score between 25 and 29 (95th percentiles) describes the risk of ON [9].

(b) The ORTO-15 [3] is a brief scale assessing orthorexia behaviors and attitudes that was initially developed and validated among Italian college students. It includes 3 subscales: cognitive aspects, clinical concerns, and emotional factors. It can be used as a total score reflecting global orthorexic tendencies with scores ranging from 15 to 60. A cutoff score of 40 was originally identified as reflecting the presence of ON. Alpha’s Cronbach in this study was 0.808.

Then, criterion validity was evaluated measuring other variables which has been demonstrated to be associated, or somehow overlapping, with ON [4, 5], such as eating disorders, depression, and obsessive–compulsive symptoms, using the following self-reported questionnaires.

(c) The Disordered Eating Questionnaire (DEQ) [22] is a 24-item scale assessing disordered eating-related behaviors and attitudes. This scale allows to calculate a valid and reliable global score of disordered eating-related behaviors (restrictive eating, binge eating and purging behaviors, willing to lose weight, ruminating, and worrying about weight and body shape, engaging in intense physical exercise to lose weight, etc.), which clinical cutoff score has been indicated as 30 [23]. Moreover, two items assessing participants’ height and weight allow us to calculate BMI (weight kg/ height m^2^). Cronbach’s α in the validation study was 0.90, while in this study was 0.933 indicating an excellent internal consistency.

(d) The Beck Depression Inventory-II (BDI-II) [24] is a 21-item self-report scale that assesses the presence and severity of affective, cognitive, and physical components of depression. The Italian version of the BDI-II showed excellent psychometric properties [25, 26]. In the present study, the Cronbach’s α was 0.896 indicating a strong internal consistency.

(e) The Obsessive Compulsive Inventory-Revised (OCI-R) [27] in the Italian version of Sica and colleagues [28] is a widely used 18-item self-report questionnaire that assesses the severity of obsessive and compulsive symptoms (washing, obsessing, hoarding, ordering, checking, and mental neutralizing). The Italian version of the OCI-R indicates good internal consistency and 30-day test–retest reliability (from 0.87 to 0.99) as well as good convergent, divergent, and simultaneous validity. In the present study, the Cronbach’s α was 0.892 indicating a strong internal consistency.

### Statistical analyses

The statistical analyses were performed using IBM SPSS software version 23 and MPLUS 8.

All I-DOS items were examined for violations of normality. Specifically, according to Tabachnick and Fidell [29], absolute skewness and kurtosis values greater than |1| reflect normality deviations. Values of several items were above the recommended cutoffs, so the analyzed variables were not realistically normally distributed. Regarding missing values, the missing rates ranged from 1.9 to 3.3% and using Little’s MCAR test [30] we highlighted that the missing pattern was missing completely at random, χ^2^ = 39.335 (p > 0.05). Given this, in MPLUS, we used the full information maximum likelihood approach (FIML), which produces unbiased parameter estimates and standard errors under MAR and MCAR [31].

With the aim of testing the original latent structure of DOS [8], a confirmatory factor analysis (CFA) model positing one-factor was carried out. Specifically, I-DOS items have only four response options, and they are not normally distributed; therefore, we treated the data as ordinal (option “categorical” in Mplus). Accordingly, model parameters were estimated using the robust weighted least squares—means and variance adjusted (WLSMV) estimator [32]. The following fit indices with respective recommended cutoff values [33] were reported: Root Mean Square Error of Approximation (RMSEA; less than 0.08 indicates an acceptable fit) with associated confidence interval and with the test of close fit that examines the probability that the approximation error is low (*p* values > 0.05 indicates a good fit); Tucker–Lewis Index (TLI; greater than 0.90 indicates an acceptable fit); Comparative Fit Index (CFI; greater than 0.90 indicates an acceptable fit); Standardized Root Mean Square Residual (SRMR; less than 0.08 indicates an acceptable fit). In addition, chi-square statistics were also reported. However, chi-square results were not considered in interpreting model fit due to its sensitivity to large sample size [34].

In the next step, according with the Meredith’s framework [35], factorial invariance tests across gender were computed by means of a hierarchical series of multigroup CFAs. With the aim to examine the latent means differences, the following levels of measurements invariance were examined: configural (i.e., same number of factors and same loading patterns across groups), metric (i.e., factor loadings equal across groups) and scalar (i.e., equivalence of item intercepts). To compare these nested models fit, chi-square difference tests were computed. In addition, difference in CFI were calculated where an ΔCFI > 0.01 indicates a significant change in model fit [34]. To check the source of lack of equivalence, MPLUS modification indices were also investigated. In this regard, when a constraint is untenable, it can be relaxed to obtain partial invariance [36].

Afterwards, internal consistency of I-DOS was evaluated by calculating the Cronbach Alpha coefficient. According to Nunnally [37], a 0.70 or above Cronbach’s alpha indicates an acceptable value.

Moreover, with the aim of testing I-DOS criterion validity, Pearson’s correlations with the ORTO-15 total score, DEQ total score, OCI-R total score, and BDI-II sum score were calculated. In addition, association between DOS and BMI was also evaluated.

## Results

### Characteristics of the sample

The sample consisted of 422 Italian students, 303 women (71.8%) and 119 men (28.2%). The mean age of participants was 20.70 years old (± 3.44), the minimum age was 18 years. The mean body mass index (BMI), based on the self-reported weight and height, was 21.83 (± 3.42). Participants who had a high school diploma were mainly represented in our sample (60.3%). Finally, only 46.3% of the sample performed physical activity, while 53.7% did not engage in any sport activity. Table 1 describes clinical variables scores evaluated in our sample (i.e., DOS, ORTO-15, DEQ, BDI-II, and OCI-R) by descriptive statistics such as mean and standard deviation.

**Table 1:** Descriptive statistics and internal consistency of the measures used assessing orthorexia nervosa (DOS and ORTO-15), disordered eating (DEQ), depression (BDI-II), and obsessive and compulsive symptoms (OCI-R)

Variable	M	SD	Cronbach’s alpha
DOS	15.60	5.35	0.888
ORTO-15	38.32	3.96	0.808
DEQ	24.17	20.47	0.933
BDI-II	10.51	9.14	0.896
OCI-R	10.06	9.73	0.892

### Confirmatory factor analysis

CFA with 415 participants was conducted for the one-factor model. Seven cases with missing values on all the measured items were not included in the analysis.

The CFA yielded ambiguous results: χ^2^(35) = 179.212, *p* < 0.001; RMSEA = 0.100, 90% CI = 0.085–0.114, *p* < 0.001; CFI = 0.970; TLI = 0.962; SRMR = 0.054). Specifically, RMSEA was widely above acceptable thresholds. Thus, we examined potential sources for this not acceptable model fit and found that two error covariance (item 6 and 10; item 4 and 7) had large and significant MI value. Both pairs of items have a similar meaning and measure similar aspect of the ON construct: in particular item 6 and 10 refer to the consequences of unhealthy eating, while item 4 and 7 refer to the social consequences of orthorexia nervosa. Accordingly, we re-ran a model with this two error covariances freely estimated. The revised model showed the following fit indices: χ^2^(33) = 112.565, *p* < 0.001; RMSEA = 0.076, 90% CI = 0.061–0.092, *p* = 0.003; CFI = 0.984; TLI = 0.978; SRMR = 0.043.

Table 2 shows the standardized factor loadings that were all above 0.70. All the factor loadings resulted statistically significant (*p* < 0.001).

**Table 2:** Confirmatory factor analysis and internal consistency results

	Standardized factor loadings	α if item is deleted	Item total correlations
1. Eating healthy food is more important to me than indulgence/enjoying the food	0.732	0.877	0.631
2. I have certain nutrition rules that I adhere to	0.790	0.878	0.644
3. I can only enjoy eating foods considered healthy	0.820	0.875	0.671
4. I try to avoid getting invited over to friends for dinner if I know that they do not pay attention to healthy nutrition	0.768	0.881	0.582
5. I like that I pay more attention to healthy nutrition than other people	0.834	0.873	0.695
6. If I eat something I consider unhealthy, I feel really bad	0.713	0.877	0.639
7. I have the feeling of being excluded by my friends and colleagues due to my strict nutrition rules	0.723	0.889	0.457
8. My thoughts constantly revolve around healthy nutrition and I organize my day around it	0.911	0.868	0.763
9. I find it difficult to go against my personal dietary rules	0.842	0.874	0.676
10. I feel upset after eating unhealthy foods	0.716	0.879	0.598

All the factor loadings are standardized and statistically significant (*p* < 0.001)

### Tests of gender factorial invariance and of gender differences

According to the revised model, factorial invariance tests across gender were examined. Invariance results are shown in Table 3.

**Table 3:** Results of the measurement invariance tests

Models	ΔCHI	CFI	ΔCFI	TLI	SRMR	RMSEA and 90% confidence interval, *p*(RMSEA < 0.05)
Configural invariance		0.990		0.986	0.053	0.064 (0.046–0.081), *p* = 0.102
Partial metric invariance	6.484 (*p* > 0.05)	0.990	0	0.987	0.054	0.061 (0.044–0.078), *p* = 0.138
Partial scalar invariance	15.769 (*p* > 0.05)	0.990	0	0.990	0.055	0.054 (0.036–0.070). *p* = 0.341

The first level (i.e., configural invariance) was achieved with the following fit indices: χ^2^(66) = 121.474, *p* < 0.001; RMSEA = 0.066, 90% CI = 0.046–0.081, *p* = 0.102; CFI = 0.990; TLI = 0.986; *SRMR* = 0.053.

When constraints on loadings were introduced, an inspection of the MI revealed that there were three constraints not tenable (factor loadings on item 7, item 1, and item 9). After they were relaxed, a partial metric invariance model was achieved (ΔCHI = 6.484, *p* > 0.05; ΔCFI = 0).

When scalar invariance model was tested, an examination of the MI revealed that introduced constraints were not tenable (thresholds of item 6, item 4, and item 10). These constraints were relaxed and a partial scalar invariance model was obtained (ΔCHI = 15.769, *p* > 0.05; ΔCFI = 0). Given all of the above, the latent means difference across gender was examined. To achieve this, mean value was constrained to zero for the male group (i.e., reference group), while in the female group was freely estimated. The results highlighted a nonsignificant difference (*p* > 0.05).

### Validity and reliability

The reliability of the I-DOS, estimated by Cronbach’s α, was 0.888, showing a strong internal consistency. Moreover, all the items showed a moderate or high correlation with the total items ranged from 0.457 to 0.763 (Table 2).

The I-DOS total score had strong and statistically significant correlations with ORTO-15 total score (r = − 0.573; *p* < 0.001), where lower ORTO-15 score indicated higher levels of orthorexia tendencies and behaviors. Significant correlations were also found with disordered eating symptoms (DEQ total score, r = 0.597; *p* < 0.001), with obsessive and compulsive symptoms (OCI-R total score, r = 0.229; *p* < 0.001), and with the sum score of depressive symptoms (BDI-II total score, r = 0.262; *p* < 0.001). Regarding the association between BMI and orthorexic eating behavior (I-DOS total score), we found a statistically nonsignificant correlation (r = 0.079, *p* > 0.05).

Table 4 presents the correlations between the I-DOS and other constructs.

**Table 4:** Correlations between orthorexia nervosa (DOS and ORTO-15), disordered eating (DEQ), depression (BDI-II), obsessive and compulsive symptoms (OCI-R), and body mass index (BMI) total scores

	ORTO-15	DEQ	BDI-II	OCI-R	BMI
I-DOS	− 0.573**	0.597**	0.262**	0.229**	0.079

**p < 0.001.

### Distribution of an estimate of orthorexia nervosa in our sample

Participants mean score of the I-DOS was 15.60 (± 5.35), scores ranging from a minimum value of 10 and a maximum value of 37. Using the original version’s cutoff points [9], 3.2% of the study participants would be considered having ON (total score greater than 30), 4.9% would be at risk of ON (total score between 25 and 29), while no risk of ON was observed in 91.9% of the sample (total score less than 25).

## Discussion

The aim of the present study was to examine the psychometric properties of the Italian translation of the DOS (I-DOS) in the Italian cultural setting. Besides, the study also explored construct validity by examining I-DOS total score correlations with different psychopathology indicators (i.e., depression, eating disorders, and obsessive and compulsive symptoms).

A CFA was performed to test the DOS’s unidimensional structure following the original creators of the questionnaire [9], with initial results revealing a questionable model fit. Subsequently, a significant improvement of the model fit was achieved after examining modification indices and an one-factor structure was observed, consistently with the previous results from the validation of DOS in other European cultures (e.g., Spain, Portugal) [15, 16]. More specifically, all fit indices of the model became acceptable after correlating error covariances of items 4 and 7 and of items 6 and 10. When examining item 4 (“I try to avoid getting invited over to friends for dinner if I know that they do not pay attention to healthy nutrition”) and 7 (“I have the feeling of being excluded by my friends and colleagues due to my strict nutrition rules''), one might discuss on the appropriateness of the language translation of these statements since no previous evidence in literature exists on high correlations between them. Nevertheless, in terms of their conceptual meaning, it may be recognized an intrinsically common theme concerning the social distress and isolation, which are crucial aspects of orthorexia assessed by the DOS [9, 13]. Regarding items 6 (“If I eat something I consider unhealthy, I feel really bad”) and 10 (“I feel upset after eating unhealthy foods”), both refer to negative feelings experienced as a consequence of eating foods considered unhealthy and previous authors [16] suggested to correlate their error covariances in order to improve the model fit. These findings are relevant insofar they explain the high correlations observed between these items and the decision to freely estimate their relative error covariance. Future studies would further explore the aforementioned item contents and eventually identify which item in the pair would be identified as conceptually redundant [38]. In this regard, in the present study, the high scale’s internal consistency (α = 0.888) and the moderated to high item-total correlations indicated robustness of the indicators.

However, in the final model obtained, different fit indices showed different acceptance levels. Specifically, SRMR, TLI and CFI were consistent with recommended cutoff values [33], whereas the RMSEA was above the cutoff. This discrepancy may depend on the fit indices used, as previous authors suggested [39]. More specifically, differently from CFI, the RMSEA is not influenced by the target-to-null models chi-square difference, but only by the target-model chi-square. When the difference among the target and the null model chi-squares is high and the target model chi-square is high, the CFI may evidence good fit, while the RMSEA does not [39]. This scenario seems to reflect the results of the present study, with the ratio of the null to target chi-square equal to 43.548, and the difference among the null and target chi-squares equal to 4789.424. Furthermore, RMSEA is also affected by model’s degrees of freedom (DFs), with DFs relatively small being associated with a larger RMSEA [39]. However, DFs of the final model were equal to 45, thus suggesting that low DFs may not be the origin of misfit, which is likely to depend on high chi-square values due to the larger sample size [39].

The results of the validity analyses revealed significant correlation coefficients between the total score of I-DOS and all the other measures included, except for BMI. First, the significant negative association found with ORTO-15, consistently with previous evidence [40], suggests a good convergent validity of the I-DOS as lower scores of ORTO-15 indicate high risk of ON [9]. On the other hand, results showed that I-DOS total score was positively related to overall eating disorder psychopathology (i.e., DEQ) as previous authors demonstrated [40]. These findings revealed that the construct of ON assessed by the I-DOS is not clearly distinguishable from the risk of eating disturbances as measured by the DEQ in the present sample. This lends support to research reporting significant relationships between orthorexic traits and levels of eating pathology [41, 42]. Generally, symptoms of ON and of eating disorders might be considerably overlapping, since healthy eating intentions and concerns about caloric intake are typically linked, especially for restrained eaters [43]. This association could be partially explained by the fact that individuals with orthorexic tendencies often report similarities with traditional eating disorders, such as the cognitive fixation on nutrition and the rigid reduction of foods considered dangerous for health or body image [44]. In addition, people with orthorexic traits were found to report a distorted perception and evaluation of their body [8], which has been regarded as a peculiar core symptom of eating disorders [2]. Overall, these findings suggest that although theoretically distinguishable, ON and eating disorders may be significantly related. Future studies are needed in order to establish more defining reliable and valid diagnostic criteria for ON.

The positive and significant correlation between I-DOS and overall depression symptomatology measured through the BDI-II found in the present study is consistent with the existing literature on the association between ON and depressive symptoms [45, 46]. Furthermore, this correlation was relatively low, thus supporting the discriminant validity of the I-DOS. Low, but significant correlation coefficient was also found between I-DOS and the severity of obsessive and compulsive symptoms measured by OCI-R total [25], consistently with the previous evidence [47]. This finding reveals that the two conditions may have similar cognitive and behavioral characteristics, as some authors suggest [48]. For example, individuals with ON spend most of their time in ritualistic behaviors and excessive efforts to select and prepare healthy food, similar to patients with obsessive and compulsive disorder (OCD) [49]. Moreover, ON is typically characterized by obsessions (e.g., overthinking about food preparation, inflated concern over contamination, and impurity) and impaired social functions like OCD [50]. These similarities between spectrum of symptoms of ON and of OCD have prompted debate as to whether orthorexia is a unique disorder or a subset of OCD [51]. Some authors suggest that the association between obsessive and compulsive tendencies and ON may reflect the high comorbidity between EDs and OCD instead to indicate ON as a disorder on the obsessive and compulsive spectrum [48]. Although further studies are needed to conclusively understand whether ON is a distinguishable pathological entity from obsessive and compulsive disease or not, we may be confident that ON is set of symptoms that are related, but distinguishable from OCD. In fact, the size of the correlation coefficients are small (according to Cohen’s categories); thus, suggesting that they are different but related constructs.

Finally, a nonsignificant association between I-DOS and BMI was observed in the present investigation. This result might indicate that ON is unrelated to weight, as previous studies demonstrated [16, 52].

In order to eventually estimate prevalence of ON assessed by the DOS, we used original cutoff points and obtained an ON prevalence of 3.2% and an ON-prevalence risk of 4.9% thus summing up to 8.1%. These results are similar to other reported in previous literature. In particular, some authors examining ON in adult samples reported comparable prevalence [53] and prevalence risk [54] percentages while others [46] found higher prevalence rates (e.g., 6.9%). These differences could be explained by the methodological approach of data collection used in the aforementioned studies. More specifically, Luck-Sikorski and colleagues [46] assessed orthorexic behaviors through a population-based telephone survey which is known to present biases related to social desirability [55]. As one of the strongest underlying motivations for ON is social desirability (i.e., being healthy to gain social support) [56], it could be hypothesized that participants of Luck-Sikorski and colleagues’ study might report exaggerated ON symptoms when interviewed by telephone, in order to appear compliant with healthy diet. Further studies are needed to examine whether different survey methods (i.e., online, offline, and telephone) for measuring ON could induce different pressure for socially desirable responding. Moreover, our prevalence percentages were estimated on the bases of German cutoff scores. Future studies should evaluate ON cases and noncases through independent instruments (e.g., a clinical evaluation) and compute cutoff scores appropriate for the Italian version of the DOS through ROC curves.

The current study clearly presents some limitations. First, its cross-sectional nature. Further studies are needed to more deeply examine the longitudinal validity of I-DOS (e.g., test–retest reliability). Second, the mere use of self-report questionnaires may be subject to social desirability effects and recall bias. Future studies should include measure of eating behavior and habits (e.g., food diary) [57] as well objective anthropometric measure and biomedical parameters. Third, the selection of nonclinical sample may limit the generalizability of our results. Future studies should evaluate the psychometric properties of the I-DOS scale in clinical samples (e.g., eating disorder or OCD patients). Finally, although our study is similar to most studies conducted in the Italian population, namely involving undergraduates and young adults, data regarding a wide age range are needed for exploring the prevalence of ON in specific stages of the life span.

## Conclusions and clinical implications

The present study aimed at adapting the Italian version of the Düsseldorf Orthorexia Scale (I-DOS) in a nonclinical sample of university students. This 10-items scale appears to be a valid and reliable measure to assess orthorexic behaviors and attitudes. The brevity of this scale and its good psychometric properties suggest that it could be a useful instrument in detecting or preventing orthorexia risk in nonclinical samples. Future studies should evaluate further psychometric characteristics and its potential use in clinical settings.

Our study gives also several suggestions regarding the construct of ON. Namely speculating about the clinical significance of the correlations coefficients between I-DOS orthorexia scores and scores on eating and obsessive disorders symptoms, consistently with an increasing amount of findings, we may hypothesize, that: (1) ON is an independent clinical entity; (2) it should be included within the eating disorders chapter of the DSM; (3) although sharing some similarities with obsessive–compulsive symptoms, it could be considered a related, but independent construct.

## What is already known on this subject?

The Düsseldorf Orthorexia Scale (DOS) is a reliable and valid instrument to assess orthorexia nervosa. It was adapted in different languages and its good psychometric properties confirmed.

## What does this study add?

This study demonstrates that the Italian version of the DOS (the I-DOS) is a valid and reliable measure to assess orthorexic behaviors and attitudes in a nonclinical sample of university students. Its original unidimensional structure has been confirmed in the Italian version with acceptable or great fit indices and a strong internal consistency.

## Data Availability

The datasets generated during and/or analysed during the current study are available from the corresponding author on reasonable request.
